# Isolation, biochemical characterization, and cloning of a bacteriocin from the poultry-associated *Staphylococcus aureus* strain CH-91

**DOI:** 10.1007/s00253-012-4578-y

**Published:** 2012-11-30

**Authors:** Benedykt Wladyka, Katarzyna Wielebska, Marcin Wloka, Oliwia Bochenska, Grzegorz Dubin, Adam Dubin, Pawel Mak

**Affiliations:** 1 Department of Analytical Biochemistry, Faculty of Biochemistry, Biophysics and Biotechnology, Jagiellonian University, Gronostajowa 7, 30-387 Krakow, Poland; 2 Department of Microbiology and Malopolska Centre of Biotechnology, Faculty of Biochemistry, Biophysics and Biotechnology, Jagiellonian University, Gronostajowa 7, 30-387 Krakow, Poland

**Keywords:** Allelic variant, Bacteriocin, Lantibiotic, *Staphylococcus aureus*, Pathogen, StpC cysteine peptidase

## Abstract

*Staphylococcus aureus* strain CH-91, isolated from a broiler chicken with atopic dermatitis, has a highly proteolytic phenotype that is correlated with the disease. We describe the isolation and biochemical and molecular characterization of the AI-type lantibiotic BacCH91 from *S*. *aureus* CH-91 culture medium. The bacteriocin was purified using a three-stage procedure comprising precipitation with ammonium sulfate, extraction with organic solvents, and reversed-phase HPLC. The BacCH91 peptide is thermostable and highly resistant to cleavage by both prokaryotic and eukaryotic peptidases. The MIC for the Gram-positive bacteria ranged from 2.5 nM for *Microococcus luteus* through 1.3–6.0 μM for staphylococcal strains up to more than 100 μM for *Lactococcus lactis*. BacCH91 was ineffective against the Gram-negative strains tested at the maximal concentration (100 μM). The amino acid sequence of BacCH91 is similar to that of epidermin and gallidermin. The encoding gene (*bacCH91*) occurred in two allelic variants distinguishable in the restriction fragment length polymorphism assay. Variant I, identified in *S*. *aureus* CH-91, dominated in *S*. *aureus* strains of poultry origin, although strains with variant II were also identified in this group. *S*. *aureus* strains of human origin were characterized exclusively by variant II.

**Electronic supplementary material:**

The online version of this article (doi:10.1007/s00253-012-4578-y) contains supplementary material, which is available to authorized users.

## Introduction

*Staphylococcus aureus* is a common opportunistic pathogen of humans and other warm-blooded animals. The bacterium is responsible for a variety of infections, constituting an important veterinary and public health issue associated with a substantial economic burden. The increasing antibiotic resistance of this bacterium is of major concern and has prompted studies on its pathophysiology.

Despite their broad host diversity, adaptation of individual strains to particular hosts occurring by acquisition of host-specific gene pools and/or loss of dispensable genetic material has been suggested in recent studies (Herron-Olson et al. 2007; Lowder et al. 2009). *S*. *aureus* strain CH-91 was initially isolated from a broiler chicken with atopic dermatitis (Kuramasu et al. 1967). The disease was correlated with a highly proteolytic phenotype (Takeuchi et al. 2002) associated with a 17-kb plasmid (pCH-91) that is homologous to the poultry-associated pAvX plasmid (Lowder et al. 2009). Both plasmids encode cysteine peptidase staphopain C (StpC), an avian host-specific virulence factor (Wladyka et al. 2011a).

Bacteria produce a wide variety of bioactive peptides, which among many other factors facilitate their environmental success. One of the important functions of bioactive peptides is the modulation of interactions with co-occurring organisms, most importantly the host and potential competitors. Staphylococci secrete a wide range of peptides involved in deregulation of the host immune response (Dinges et al. 2000; Foster 2005; Postma et al. 2004). The range of secreted peptides varies among strains, and some profiles correlate with a virulent phenotype (Wang et al. 2007). Bacteria produce a diverse range of polypeptides involved in both inter- and intra-species interactions. Among these, bacteriocins are peptides directed against a variety of bacteria; hence, they are of special interest, given their potential use in medicine and the food industry (Galvez et al. 2007; Gillor and Ghazaryan 2007). Staphylococci provide a rich source of diverse bacteriocins. To date, five such peptides (Pep5, epidermin, epicidin 280, epilancin K7, and epidermicin NI01) have been characterized from *Staphylococcus epidermidis* (Heidrich et al. 1998; Kaletta et al. 1989; Sandiford and Upton 2011; Schnell et al. 1988; van de Kamp et al. 1995). Among other bacteriocins, gallidermin was isolated from *Staphylococcus gallinarum* (Kellner et al. 1988), hominicin was isolated from *Staphylococcus hominis* (Kim et al. 2010), and nukacin ISK-1 was isolated from *Staphylococcus warneri* (Sashihara et al. 2000). Strains of *S*. *aureus* have also been shown to produce bacteriocins, including the two-peptide lantibiotic staphylococcin C55 (Navaratna et al. 1998), the closely related staphylococcin BacR1 (Crupper et al. 1997), the tryptophan-rich aureocin A53 (Netz et al. 2002, 2001), and aureocin A70. Contradictory reports exist concerning the presence of epidermin homologues in *S*. *aureus* (Daly et al. 2010; Joo et al. 2011). Intriguingly, with the exception of gallidermin, the genes encoding the bacteriocins noted above are located on plasmids, often together with genes determining antibiotic resistance or encoding virulence factors.

Here, we describe the isolation and biochemical characterization of bacteriocin BacCH91 from *S*. *aureus* strain CH-91 and show for the first time the ability of *S*. *aureus* to produce the epidermin-like lantibiotic. Cloning of the encoding gene *bacCH91* and a comparative sequence analysis facilitated identification of a unique allelic variant of *bacCH91* associated with *S*. *aureus* strains originating from poultry.

## Materials and methods

### Bacterial strains and growth conditions

*S*. *aureus* CH-91 was kindly provided by S. Takeuchi from Fukui Prefectural University, Japan (Takeuchi et al. 1999). The strain has been deposited in the DSMZ collection under the number DSM 26258. *Bacillus licheniformis* NCTC 7224, *Bacillus subtilis* ATCC 6633, *Lactococcus lactis* subsp. *lactis* LOCK 0869, *Micrococcus luteus* strains DSMZ 20030 and CBM (clinical isolate), *Sarcina lutea* ATCC 9341, *S*. *aureus* strains ATCC 25923, M-122 (Polakowska et al. 2012), Newman and RN4220, *S*. *epidermidis* ATCC 35347, *Staphylococcus intermedius* ATCC 29666, *Staphylococcus pseudintermedius* LMG 22219 (Devriese et al. 2005), *Staphylococcus saprophyticus* ATCC 49453, *Staphylococcus xylosus* strains isolated from the skin of healthy chickens, *Streptococcus mutans* ATCC 25175, *Streptococcus pneumoniae* ATCC 49619, *Escherichia coli* K12 ATCC 10798, *Klebsiella pneumoniae*, *Serratia marcescens*, and *Sphingomonas* sp. AMC 3962 (Dziga et al. 2012) were obtained from the collections of The Faculty of Biochemistry, Biophysics and Biotechnology, Jagiellonian University, Krakow, Poland. A list of strains used for assessment of the general occurrence of the bacteriocin gene is provided in the Electronic supplementary materials (ESM; Table S1). Gram-positive and Gram-negative bacteria were maintained at 37 °C in tryptic soy broth (TSB) and Luria-Bertani broth (both obtained from Sigma), respectively. To determine the minimal inhibitory concentration (MIC) doses, all bacteria were cultivated in cation-adjusted Mueller-Hinton broth (MHB, Sigma) or, in the case of *Streptococcus* spp. and *L*. *lactis*, grown on agar-solidified MHB supplemented with 2.5 % lysed horse blood (LHB, Graso).

### Purification of bacteriocin

Bacteriocin BacCH91 was purified from *S*. *aureus* CH-91 culture supernatant. The bacteria were cultivated in TSB at 37 °C and 200 rpm shaking for 18 h. The culture was centrifuged at 17,000×*g* for 15 min at 4 °C. The supernatant was acidified to pH 3.0 using 5 M HCl and then precipitated with ammonium sulfate to 80 % saturation at 4 °C. The precipitated material was recovered by centrifugation at 17,000×*g* for 30 min at 4 °C. The pellet was dissolved in water and freeze-dried. The resulting dry powder was extracted for 5 min in a mixture of chloroform and methanol (2:1, *v*/*v*) with vigorous shaking at room temperature (RT). The extract was clarified by centrifugation (13,000×*g* for 5 min at RT). The clear supernatant was removed and dried in a centrifugal evaporator. The dry extract was dissolved in water containing 27 % (*v*/*v*) acetonitrile and 0.09 % (*v*/*v*) trifluoroacetic acid (TFA), and the resulting preparation was filtered through a 0.45-μm centrifugal filter and subjected to high-performance liquid chromatography (HPLC) separation on a LC-8 column (150 × 4.6 mm; 3 μm particle size; Supelco) using a buffer system comprising buffers A (0.1 % TFA; *v*/*v*) and B (0.07 % TFA in 80 % acetonitrile; *v*/*v*), with a linear gradient from 40 to 80 % buffer B over 20 min at 1 ml/min. Fractions containing BacCH91 were detected by measuring absorbance at 220 and 280 nm, collected, dried in a centrifugal evaporator, dissolved in water, and stored at −20 °C until further use.

### Protein chemistry techniques

Chemical derivatization to enable detection of modified amino acids in the bacteriocin molecule during N-terminal sequencing was performed according to Meyer et al. (1994). In brief, water was evaporated from the BacCH91 solution in a centrifugal evaporator. The bacteriocin was re-dissolved in a solution containing 46 % ethanol (*v*/*v*), 10 % ethanethiol (*v*/*v*), and 0.5 M NaOH. The preparation was purged with argon and sealed. Following incubation for 2 h at 50 °C, the preparation was acidified with glacial acetic acid, vortexed, and used for N-terminal amino acid sequencing.

The N-terminal amino acid sequence was determined in an automatic protein sequencer (Procise 491; Applied Biosystems) using Edman degradation of the polypeptide chains. The polybrene-coated glass fiber discs were used for sample immobilization according to the manufacturer’s instructions.

Amino acid analysis was performed according to White et al. (1986). In brief, bacteriocin was hydrolyzed in the gas phase over 6 M HCl at 115 °C for 24 h. The liberated amino acids were converted to phenylthiocarbamyl derivatives and analyzed by HPLC (PicoTag 150 × 3.9 mm column; Waters). The total protein concentration was estimated using the bicinchoninic acid assay (BCA, Sigma).

Electrospray ionization mass spectrometry (ESI-MS) was carried out using an HCT Ultra mass spectrometer (Bruker) equipped with an electrospray source and an ion trap analyzer.

Tris-tricine sodium dodecyl sulfate polyacrylamide gel electrophoresis (SDS–PAGE) was carried out under reducing conditions using peptide-separating gels, according to the protocol of Schagger and von Jagow (1987). Following electrophoresis, the gels were fixed for 30 min in a mixture of 50 % methanol (*v*/*v*) and 10 % acetic acid (*v*/*v*), and stained with Coomassie Brilliant Blue R-250.

### Determination of MIC doses

The bactericidal activity of BacCH91 was determined using the micro-dilution method in compliance with Clinical and Laboratory Standards Institute guidelines M7-A7 (CLSI 2006). Briefly, the bacteria were either cultivated in cation-adjusted MHB to the early exponential phase or grown overnight on agar-solidified MHB supplemented with 2.5 % LHB in the case of *Streptococcus* spp. and *L*. *lactis*. Subsequently, the bacteria were diluted to 1 × 10^6^ CFU/ml either in MHB or in MHB supplemented with 5 % LHB, in the case of the latter bacteria. Fifty-microliter portions of bacterial suspension were mixed with an equal volume of MHB containing serial twofold dilutions of bacteriocin. After overnight incubation at 37 °C, the optical density (OD) at 600 nm was measured. The MIC was defined as the minimum bacteriocin concentration for which the increase in OD was not observed compared to uninoculated broth.

### Radial diffusion assay

For pH, temperature, and protease resistance assays, the residual activity of BacCH91 was determined using the radial diffusion assay (Lemos Miguel et al. 2008). In brief, plates were prepared containing 200× diluted overnight culture of *S*. *lutea* ATCC 9341 in TSB agar (1 %, *w*/*v*). Wells (2.4 mm diameter) were cut in the agar and filled with 10 μl of the BacCH91 preparation for testing. Following overnight incubation at 37 °C, the residual activity was measured as the diameter of inhibition zones.

### Influence of pH, temperature, and proteases on the activity of BacCH91

To test the pH stability of BacCH91, a water solution of the bacteriocin (final concentration, 30 μM) was evaporated in a vacuum centrifuge and re-dissolved in (a) 100 mM sodium citrate pH 3.0, 4.0, 5.0, and 6.0; (b) 100 mM Tris–HCl pH 7.0, 8.0, and 9.0; and (c) 100 mM 3-cyclohexylamino-1-propanesulphonic acid-NaOH pH 10.0 and 11.0. The solutions were incubated for 3 h at RT, and the residual activity of the bacteriocin was determined using the radial diffusion assay. The temperature stability of BacCH91 (30 μM final concentration) was determined in 50 mM Tris–HCl pH 7.4, 0.15 M NaCl and 50 mM bis-Tris pH 6.4, 0.15 M NaCl. The solutions were incubated for 1, 6, and 24 h at 37, 60, 80, and 99 °C. The residual activity of the bacteriocin was determined with the radial diffusion assay using *S*. *lutea* as an indicator strain.

We evaluated the susceptibility of BacCH91 to proteolysis by aureolysin; staphopains A, B, and C; V8 peptidase (Biocentrum); bovine alpha-chymotrypsin and TPCK-treated trypsin (Sigma); human elastase and cathepsin G (Biocentrum); pepsin from porcine gastric mucosa (Sigma); and proteinase K (A&A Biotechnology). Dried samples of BacCH91 were dissolved to a final concentration of 30 μM in the following buffers: 100 mM bis-Tris pH 6.4 or Tris–HCl pH 7.5, each containing 5 mM CaCl_2_ (aureolysin, chymotrypsin, and trypsin); 100 mM bis-Tris pH 6.4 or Tris–HCl pH 7.5, each containing 5 mM cysteine hydrochloride and 5 mM EDTA (staphopains A, B, and C); 100 mM bis-Tris pH 6.4 or Tris–HCl pH 7.5 (elastase); 100 mM bis-Tris pH 6.4 or Tris–HCl pH 7.5, each containing 200 mM NaCl (cathepsin G); 100 mM bis-Tris pH 6.4 or 100 mM sodium phosphate pH 6.4 and pH 7.8 (V8 peptidase); and 100 mM sodium citrate pH 3.2 (pepsin). The peptidases were added in the ratio 1:20 (enzyme/bacteriocin) by weight, and the mixtures were incubated for 1 h at 37 °C. The residual activity was determined with the radial diffusion assay using *S*. *lutea* as an indicator strain, and the bacteriocin molecule integrity was assessed using HPLC.

All of the above experiments were performed in triplicate. The final results are presented as means ± SD.

### Influence of StpC on biosynthesis of BacCH91

*S*. *aureus* CH-91 was cultured at 37 °C in TSB supplemented with the cysteine peptidase inhibitor E-64 (1-[*N*-[(l-3-trans-carboxyoxirane-2-carbonyl)-l-leucyl] amino]-4-guanidinobutane; Sigma) at final concentrations of 1.0, 0.1, and 0.01 mM. Aliquots were removed at 5, 10, and 24 h, and the bacteria-free supernatants were obtained by centrifugation.

To estimate the level of BacCH91, the collected supernatants were acidified with TFA (1 % final concentration, *v*/*v*) and filtered (0.45 μm filter). Aliquots (30 μl) were analyzed using HPLC (Kromasil C4 column, 250 × 4.6 mm; Supelco) and the same buffers as those used during the purification procedure. The column was flushed for 5 min following injection at a constant concentration of 34 % B. A linear gradient from 34 to 100 % B was then applied over 10 min at 1 ml/min. Under these conditions, BacCH91 was detected at 220 nm as a single peak eluting with a retention time of 14.6 min. The relative amount of bacteriocin was calculated as the area under the peak.

The residual proteolytic activity of collected culture media was determined at 37 °C using Hide Powder Azure (Calbiochem) in 0.1 M Tris–HCl (pH 7.5) supplemented with 5 mM cysteine and 5 mM EDTA.

### Determination of the bacteriocin coding sequence

The coding sequence for BacCH91 and its flanking regions was determined using standard molecular biology methods (Sambrook et al. 1989). Briefly, the amino acid sequence of bacteriocin determined using Edman sequencing was compared with sequences in the National Center for Biotechnology Information (NCBI) database (http://www.ncbi.nlm.nih.gov), using the BLASTp algorithm. Based on the matching genomic sequences obtained, we designed a set of primers flanking the region of interest. Amplicons obtained by PCR using *S*. *aureus* CH-91 genomic DNA were cloned into pTZ57R/T (Fermentas). The plasmids were sequenced across the inserts. The contig sequence was edited using Genedoc 2.0 software (Nicholas et al. 1997) and deposited in the GenBank (accession number JQ655767).

### Determination of the distribution of the bacteriocin gene

The distribution of the bacteriocin gene among the staphylococcal strains was determined using PCR. For PCR, the primers flanking the bacteriocin gene, i.e., bacteruniqueF (5′-TTAGTGAAAATAAATAGTA) and bacterrewR (5′-CATTTGTAAGCACCTCAC), were used under the following PCR cycling conditions: 1 cycle at 94 °C for 2 min, 30 cycles at 94 °C for 30 s, 42 °C for 30 s, and 72 °C for 45 s. Strains positive for *bacCH91* were further assayed to assess the occurrence of the allelic variant of *bacCH91*. The PCR product was directly treated with the restriction enzyme *BcuI* (Fermentas). The restriction fragments were separated using 2 % agarose gel electrophoresis. Restriction pattern I consisted of 271 and 128 bp products of a single cleavage, whereas restriction pattern II was characterized by an uncleaved amplicon of 399 bp.

## Results

### Purification and biochemical characterization of bacteriocin BacCH91

**Fig. 1:**
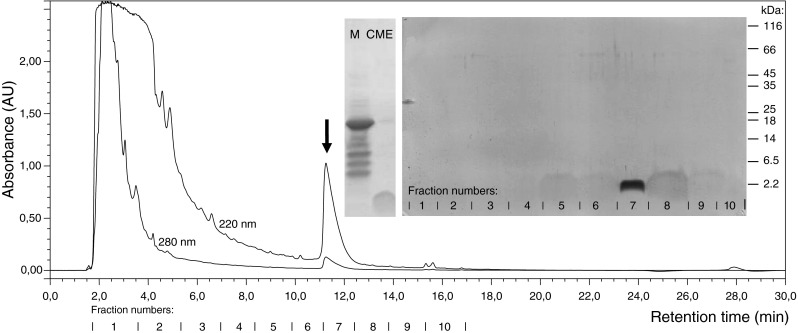
HPLC purification of BacCH91. The chloroform/methanol extract of the precipitated culture medium was separated on a C8 column. The *arrow* indicates the homogenous bacteriocin peak. The *inserts* contain the SDS–PAGE image of the culture medium (*M*), chloroform/methanol extract (*CME*), and the respective fractions from the C8 column

BacCH91 was purified from the culture supernatant of *S*. *aureus* CH-91 using a three-stage procedure comprising precipitation with ammonium sulfate, extraction with organic solvents, and reversed-phase HPLC on a C8 column. Using the optimized gradient, the bacteriocin eluted at approximately 11.5 min (Fig. 1). The preparation obtained was homogenous, as evidenced by SDS–PAGE (Fig. 1), ESI-MS, and N-terminal sequencing (data not shown). The purification procedure yielded 1 mg purified BacCH91 from 1 L of culture supernatant (10 % overall yield; Table 1).

**Table 1:** Summary of the purification process of BacCH91 bacteriocin

Stage of purification	Fraction volume (ml)	Protein concentration^a^ (mg/ml)	Total protein^a^ (mg)	Specific activity^b^ (U/mg)	Total activity^b^ (U)	Yield (%)	Purification factor (fold)
Culture supernatant	500	5.65	2,825	19.35	54,664	100	1
Ammonium sulfate precipitation	29.9	7.65	228.7	55.99	12,805	23.4	2.89
Organic extract	3.85	17.0	65.5	92.08	6,031	11.0	4.76
HPLC	1	0.548	0.548	10,220	5,600	10.2	528

^a^The protein concentration was routinely determined using the BCA. Only for the final peptide preparation (following HPLC) was the concentration measured by amino acid analysis

^b^One unit of bactericidal activity (units) was arbitrarily defined as the amount of bacteriocin resulting in an inhibition zone of 1 cm diameter, as determined in the radial diffusion assay

Based on ESI-MS, the molecular mass of the purified bacteriocin was estimated at 2,074.9 Da. Direct N-terminal Edman degradation allowed only partial determination of the first six residues: Ile-X-X-Phe-Ile-Gly, where X denotes an unknown residue. Identification of amino acid residues at positions 2, 3, 7, and subsequent positions was not possible in the standard setup, suggesting posttranslational modification of side chains or unusual intramolecular bonds, both of which commonly occur in lantibiotics. Identification of residues at positions 2, 3, 7, and subsequent positions was possible only after the chemical derivatization of the bacteriocin with ethanethiol under alkaline conditions. The full 21-mer sequence of BacCH91 was found to be Ile-bmeSeCys-SeCys-Phe-Ile-Gly-SeCys-bmeSeCys-Pro-Gly-X-Gly-Lys-bmeSeCys-Gly-SeCys-Phe-Asn-SeCys-Phe-SeCys, where X is an unknown residue, bmeSeCys is beta-methyl-S-ethylcysteine (a reaction product of ethanethiol with 2,3-didehydro-2-aminobutyric acid or 3-methyl-lanthionine), and SeCys is S-ethyl-cysteine (a reaction product of ethanethiol with 2,3-didehydroalanine or lanthionine).

### Antibacterial activity

The bactericidal activity of the purified BacCH91 was determined for a range of Gram-positive and Gram-negative bacteria using the micro-dilution assay. The bacteria tested included 11 strains of staphylococci, 7 strains of other Gram-positive bacteria, and 5 Gram-negative species (Table 2). With the exception of *L*. *lactis*, for which the determined MIC was over 100 μM, all the Gram-positive bacteria tested were susceptible to BacCH91. Among the microorganisms tested, *M*. *luteus* strains exhibited the highest susceptibility to the bacteriocin (2.5 and 40 nM for DSMZ 20030 and CBM, respectively). For other Gram-positive bacteria, the MICs ranged from a 0.31 μM for *S*. *lutea* to 6.0 μM for various *S*. *aureus* strains. Interestingly, the bacteriocin-producing strain *S*. *aureus* CH-91 was also susceptible to BacCH91 (MIC 1.3 μM). None of the Gram-negative species assayed were susceptible to BacCH91, even at the highest concentration tested (100 μM).

**Table 2:** Antibacterial activity (MIC) of BacCH91 towards selected Gram-positive and Gram-negative strains

Strain	MIC (μM)
*Bacillus licheniformis* NCTC 7224	1.8
*Bacillus subtilis* ATCC 6633	2.6
*Lactococcus lactis* subsp. *lactis* LOCK 0869	>100
*Micrococcus luteus* DSMZ 20030	0.0025
*Micrococcus luteus* CBM	0.04
*Sarcina lutea* ATCC 9341	0.31
*Staphylococcus aureus* ATCC 25923	6.0
*Staphylococcus aureus* CH91^a^	1.3
*Staphylococcus aureus* M-122	4.0
*Staphylococcus aureus* Newman^a^	6.0
*Staphylococcus aureus* RN4220^a^	6.0
*Staphylococcus epidermidis* ATCC 35347	1.6
*Staphylococcus intermedius* ATCC 29663	1.6
*Staphylococcus pseudintermedius* LMG 22219	1.5
*Staphylococcus saprophiticus* ATCC 49453	3.0
*Staphylococcus xylosus* kw 2,5^b^	1.5–2.0
*Streptococcus mutans* ATCC 25175	2.0
*Streptococcus pneumoniae* ATCC 49619	1.6
*Escherichia coli* K12 ATCC 10798	>100
*Klebsiella pneumoniae*	>100
*Pseudomonas aeruginosa* ATCC 27853	>100
*Serratia marcescens*	>100
*Sphingomonas* sp. AMC 3962	>100

^a^Bacteriocin gene positive strain

^b^Two representative strains from a single herd were tested

### Effect of pH, temperature, and peptidases on BacCH91 activity

**Fig. 2:**
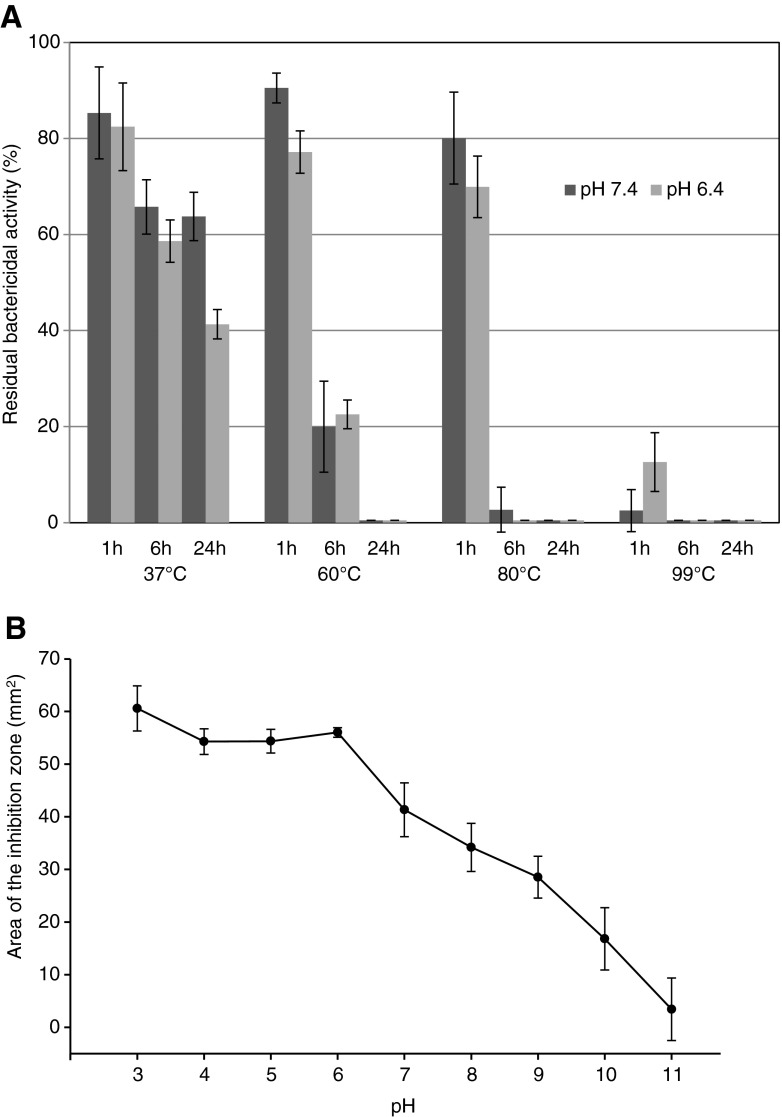
Temperature and pH stability of BacCH91. **a** Residual bactericidal activity of bacteriocin following treatment for the indicated times at the indicated temperatures and pH. **b** Residual bactericidal activity 3 h following bacteriocin exposure to buffers of the indicated pH. A radial diffusion assay with the *S*. *lutea* indicator strain was used to determine the residual bactericidal activity. For other experimental details, see “Materials and methods” section

The effect of pH on the activity of BacCH91 was studied in the range pH 3–11. Purified bacteriocin retained full bactericidal activity during incubation for 3 h at pH 3.0–6.0 (Fig. 2b). Incubation at pH 7.0 and higher pH values caused a gradual loss of bactericidal activity, and complete inactivation occurred at pH 11.0.

The effect of temperature on the activity of BacCH91 was studied at pH 6.4 and pH 7.4. The bacteriocin was incubated at 37, 60, 80, and 99 °C, and the residual activity was determined using the radial diffusion assay and *S*. *lutea* as an indicator strain (Fig. 2a). BacCH91 maintained its activity for 1 h at temperatures up to 80 °C. Even short incubation at 99 °C or several hours of incubation at temperatures higher than 60 °C resulted in complete inactivation of the bacteriocin. The temperature-induced inactivation was associated with hydrolysis of the polypeptide chain as evidenced by HPLC and ESI-MS analyses (not shown).

The susceptibility of BacCH91 to proteolysis was studied at pH 6.4 and at the optimum pH for each of the enzymes tested. Following incubation with each enzyme, the residual bactericidal activity was determined using the radial diffusion assay, and the integrity of BacCH91 was analyzed using HPLC. None of the proteases tested (see “Materials and methods” section) affected the activity or integrity of BacCH91, including all of the staphylococcal extracellular peptidases tested.

### Influence of StpC on the biosynthesis and stability of BacCH91

*S*. *aureus* strain CH-91 secretes large quantities of cysteine peptidase StpC, an enzyme that constitutes the major component of the extracellular, soluble proteome of the bacterium (Wladyka et al. 2011b). It was therefore of interest to determine the effect of the enzyme on the biosynthesis and stability of BacCH91 *in vivo*. The bacteria were cultured in liquid medium in the presence of E-64, a specific irreversible inhibitor of cysteine peptidases. Samples of the culture medium were collected after 5, 10, and 24 h, and the amount of bacteriocin was estimated by HPLC. The amount of BacCH91 was the same for all concentrations of the E-64 tested (0.01, 0.1, and 1.0 mM) and in the control medium (no inhibitor added). StpC inhibition was tested in parallel using the chromogenic protein substrate. No residual proteolytic activity was detected in the culture media containing the inhibitor, thereby confirming its effectiveness. This experiment indicated that StpC is dispensable for posttranslational maturation of BacCH91 and confirmed that the peptidase does not affect the stability of the lantibiotic.

### Characterization of the gene encoding BacCH91 and its localization in the staphylococcal genome

The sequence of BacCH91 obtained using Edman degradation was aligned with staphylococcal genomic data using the BLASTp algorithm in the NCBI database. An open reading frame (ORF) encoding an identical sequence was identified in three *S*. *aureus* strains (RF122, LGA 251, and ED133), all of which were isolated from animals. Additional matches of lesser similarity (but differing only at several positions) were identified in strains of human origin, but also in RF122, LGA 251, and ED133, indicating the presence of two genes encoding similar bacteriocins in the genomes of some *S*. *aureus* strains. The sequence encoding BacCH91 is not present in the genome of strain ED98, which was isolated from chicken, or other sequenced *S*. *aureus* genomes. This indicates that the *bacCH91* gene is not a part of the core genome but rather a part of an accessory genetic pool.

**Fig. 3:**
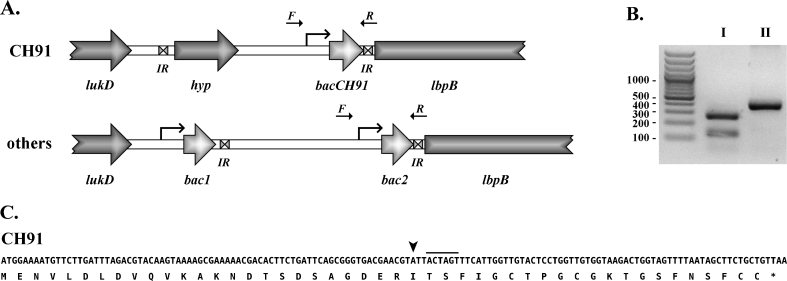
Chromosome organization in the vicinity of the *bacCH91* gene. **a** Schematic organization of the chromosome region containing the bacteriocin gene in *S*. *aureus* CH-91 and other *S*. *aureus* strains. Note: some strains were devoid of bacteriocin genes and the entire region downstream of *lukD* was organized very differently, which is not shown here. ORFs are shadowed *gray*. Putative promoter regions are indicated with the *bent arrows*. The primers used for amplification of the bacteriocin allele are indicated by *straight arrows*. Inverted repeats are indicated by “*IR*” and a *double triangle symbol*. **b** Agarose gel showing an example RFLP result for two allelic variants of the *bacCH91* (*bac2*) gene. **c** Nucleotide and amino acid sequence of the pre-peptide of BacCH91. An *arrowhead* indicates the beginning of the mature form. The *line above the sequence* indicates the cleavage site for *BcuI*, which was used for determining the allelic variants

To determine the precise location and neighborhood of *bacCH91* in the genetic material of *S*. *aureus* CH-91, a 2-kbp fragment flanking the gene was amplified and sequenced. The analysis of the obtained sequence demonstrated that the gene encoding leukotoxin D (*lukD*) was located 861 nucleotides upstream of *bacCH91*, while *lbpB*, a gene coding for a lantibiotic biosynthesis protein B, was located 64 nucleotides downstream of *bacCH91* (Fig. 3). Although the neighborhood of *bacCH91* resembles that of the other staphylococcal bacteriocins identified during this study and described in the published staphylococcal genomes, significant differences were also found. Other staphylococcal lantibiotic gene clusters encode two very similar bacteriocins, whereas only a single gene (*bacCH91*) was present in *S*. *aureus* CH-91 (Fig. 3a). Moreover, a sequence with no homology to any database sequence was found downstream of *lukD* in strain CH-91. This sequence was approximately 200 bp long and ended with 13-nucleotide inverted repeats. Downstream of this sequence in strain CH-91, instead of the *bac1* lantibiotic gene found in the other strains of staphylococci, we identified an ORF (*hyp*) that encodes an 86-amino acid polypeptide having moderate homology to the C-terminal part of the staphylococcal IstB-like ATP-binding protein followed by *bacCH91* (a *bac2* homologue). Downstream of *bacCH91*, an 11-nucleotide perfect IR identical to that found downstream of *bac2* in the other strains was identified.

### Occurrence of the lantibiotic gene allele in *S*. *aureus* strains

Analysis of available genomic data revealed that *bacCH91* homologues (*bac2*) occur in two conserved allelic variants (Fig. 3 and ESM Fig. S1). A lantibiotic gene identical to that of strain CH-91 was found in only three strains of *S*. *aureus* (RF122, LGA 251, and ED133), all of which were isolated from cows, whereas neither *bac1* nor *bac2* was present in the ED98 strain, which is of chicken origin. An allelic variant of *bac2* was identified in some strains isolated from humans (other human strains had neither *bac1* nor *bac2*). The identified alleles of *bac2* differ in the presence of a *BcuI* (*SpeI*) site, which conveniently facilitated identification using restriction fragment length polymorphism (RFLP) analysis (Fig. 3b, c). To assess the general occurrence of allelic variants, 58 *S*. *aureus* strains were tested, including 21 of human origin and 37 isolated from animals. PCR products (399 bp) containing the lantibiotic gene (Fig. 3b), obtained using the bacteruniqueF and bacterrewR primers, were treated with *BcuI*, and the restriction products were analyzed using electrophoresis. A total of 23 strains (11 of human origin and 12 of animal origin) were positive for the *bac2* gene (Table 3 and ESM Table S1). The type I restriction pattern characteristic of strain CH-91 was found in 8 of the 12 bacteriocin-positive strains isolated from animals but in none of the 11 bacteriocin-positive strains of human origin. With respect to the strains isolated from poultry, 9 of the 20 strains were positive for the lantibiotic gene, with seven strains exhibiting a type I restriction pattern. It is noteworthy that the two remaining strains with a type II restriction pattern were MRSA strains, while all the other poultry strains were MSSA.

**Table 3:** Occurrence of allelic variants of the bacteriocin gene among *S*. *aureus* strains

Strain origin	*S*. *aureus* strain		Detectable bacteriocin gene		Restriction pattern			
					I		II	
	Quantity	%	Quantity	%	Quantity	%	Quantity	%
Human	21	36.2	11	52.4	0	0	11	100
Poultry	20	34.5	9	45.0	7	77.8	2	22.2
Non-poultry	17	29.3	3	17.6	1	33.3	2	66.6
Total	58	100	23	39.7^a^	8	34.8^b^	15	65.2^b^

^a^Percent of bacteriocin gene positive strains among all tested strains

^b^Percent of the respective restriction pattern among bacteriocin gene positive strains

## Discussion

The BacCH91 reported here is the first AI-type lantibiotic isolated from *S*. *aureus* and thoroughly characterized. Existence of a gene coding for similar bacteriocin was previously reported (Daly et al. 2010), but the described properties of a corresponding peptide were questioned by Joo et al. (2011). The former authors claimed to have identified a respective peptide in culture supernatants of *S*. *aureus* based on mass spectrometry data. The identification was however doubtful (2 Da molecular weight disagreement), and afterwards, Joo and collaborators noticed that the molecular mass of the antimicrobial agent identified by Daly was in perfect agreement with proteolytically processed phenol soluble modulin, also exhibiting pronounced antimicrobial activity. Therefore, the identity of the antimicrobial agent characterized by Daly and colleagues remains uncertain.

**Fig. 4:**
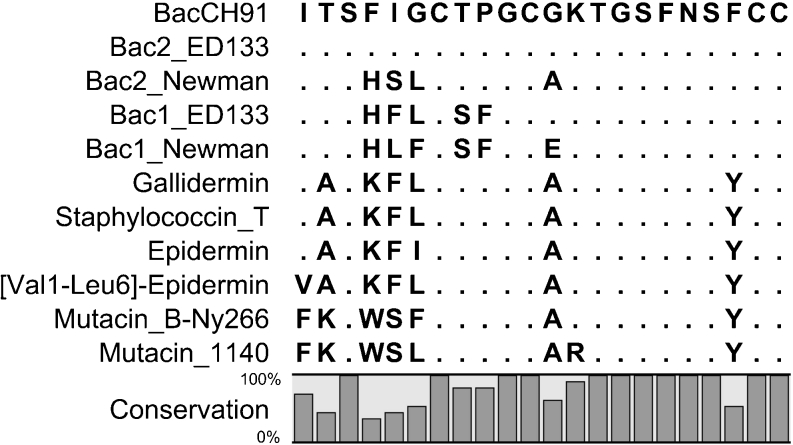
Alignment of the amino acid sequences of AI-type lantibiotics. Identical residues are indicated by *dots*

The BacCH91 bacteriocin was purified from culture supernatants of strain CH-91 using a procedure originally designed for bacterial hemolytic peptides (Mak et al. 2008). The relatively hydrophobic character of the peptide facilitated effective extraction using an optimized mixture of organic solvents, and a single subsequent chromatography step (reversed-phase HPLC) produced a homogenous bacteriocin preparation. This procedure is much less time-consuming than the procedures that have been used by others to purify similar peptides. Although the yield (10 %) was not as high as that reported for some bacteriocins (e.g., 19 % for staphylococcin T), it was still higher than that reported for other bacteriocin isolation procedures (e.g., 1 % for mutacin B-Ny266) (Furmanek et al. 1999; Mota-Meira et al. 1997). Determination of the amino acid sequence of BacCH91 using Edman degradation required chemical modification of multiple residues, as previously reported for other lantibiotics (Meyer et al. 1994). The results of chemical sequencing were confirmed by cloning of the bacteriocin gene. Based on the sequence obtained, BacCH91 was classified in the epidermin group of AI-type lantibiotics, which includes peptide epidermin; Val1-Leu6-epidermin; gallidermin; staphylococcin T; mutacins B-Ny266, 1140, I and III; and streptin (Bierbaum and Sahl 2009; Chatterjee et al. 2005). BacCH91 is most closely related to epidermin and gallidermin (Fig. 4). Significantly, the fourth residue was phenylalanine in BacCH91 but lysine in both epidermin and gallidermin, which explains the more hydrophobic character of BacCH91.

The MICs of BacCH91 for various bacterial species clearly demonstrated that the bacteriocin was effective only against Gram-positive microorganisms, however not against *L*. *lactis*. Among the species tested, *M*. *luteus* exhibited the highest susceptibility to BacCH91. The MIC determined for this species (2.5 nM for the strain DSMZ 20030 and 40 nM for the strain CBM) is comparable with the value obtained for closely related galidermin (Ottenwalder et al. 1995), which indicates that at least one likely target bacterial species has been identified. The high susceptibility of *M*. *luteus* to bacteriocin produced by *S*. *aureus* has possible biological relevance since both bacteria are residents of mammalian skin and nasal mucosa. Therefore, it is likely that the bacteriocin-producing staphylococcal strains have an advantage in competing for the same ecological niche with *Micrococcus* sp. The MIC values for *Staphylococcus* spp. did not differ significantly among species and strains, and they were most comparable to the MIC for *S*. *aureus* strain CH-91. This indicates that BacCH91 action is not directed against other, non-bacteriocin-producing staphylococcal strains.

No differences in the temperature stability of BacCH91 were observed between neutral pH and pH 6.4, which is typical of chicken skin (Bhaduri and Cottrell 2004). Similarly to other lantibiotics, the bacteriocin from *S*. *aureus* strain CH-91 was stable at elevated temperatures (almost 100 % activity was retained during incubation for 1 h at 80 °C) and resistant to proteolytic inactivation. The bacteriocin was not affected by any of the staphylococcal peptidases tested, particularly StpC, which is abundantly produced by strain CH-91. This was expected as it was assumed that secretory peptides would not be inactivated by proteases secreted by the same microorganism into the same environmental niche. Surprisingly, BacCH91 was unaffected by trypsin and proteinase K. This result differs from that obtained in previous studies of AI-type epidermin-like and nisin-like lantibiotics which are typically cleaved by trypsin, although only after prolonged incubation (Kellner et al. 1988; Kuipers et al. 1993).

Lantibiotics are ribosomally synthesized peptides that undergo extensive posttranslational modification, including proteolytic processing of the leader peptide. It has been demonstrated that as many as three extracellular peptidases (subtilisin, WprA, and Vpr) are involved in maturation of subtilin, produced by *B*. *subtilis* ATCC 6633 (Corvey et al. 2003). Other antimicrobial peptides require activation by endogenous extracellular peptidases (Faye et al. 2002). Since BacCH91 is produced by a highly proteolytic strain, it was important to assess whether StpC, the major extracellular peptidase of strain CH-91, is engaged in maturation of bacteriocin. Inhibition of StpC activity during bacterial growth using a specific irreversible inhibitor of cysteine peptidases (E-64) had no effect on the level of BacCH91, suggesting an StpC-independent maturation mechanism. This finding is consistent with the recently reported substrate specificity of StpC for bulky uncharged amino acid residues (Kalinska et al. 2012); in turn, the BacCH91 leader peptide is processed after a charged residue (Fig. 3c).

Since BacCH91 is the first AI-type lantibiotic characterized from *S*. *aureus*, we assessed whether identical or homologous lantibiotics were present in other strains. The database search revealed that a gene encoding an identical bacteriocin was present in *S*. *aureus* strains isolated from cows. Moreover, highly homologous genes were found in some strains of human origin, whereas many strains of both human and animal origin completely lacked genes homologous to *bacCH91*. Therefore, it was concluded that *bacCH91* is accessory to the core genome of staphylococci. This is of particular importance since accessory genetic material has been implicated in virulence and host specificity of staphylococci (Lowder et al. 2009). The genomic localization of *bacCH91* contrasts with other identified staphylococcal bacteriocin genes, which are all located on plasmids (Bierbaum et al. 1996). Analysis of staphylococcal genomic data demonstrated that bacteriocins identical or closely related to BacCH91 were encoded in tandem repeats (Daly et al. 2010). Only strain CH-91 exhibited unique organization of the region involved, with only a single *bacCH91* gene being present, while the second bacteriocin gene was substituted by an ORF encoding a polypeptide similar to proteins that are thought to be involved in recombination of genetic material (Solinas et al. 1995; Fig. 3a). This indirectly suggests the origin of the observed genetic rearrangement. In all the cases, the gene encoding a lantibiotic biosynthesis protein (LbpB) was located downstream of *bacCH91* (*bac2*). This organization is comparable to that of a cluster of genes driving the biosynthesis of the homologous lantibiotic epidermin, produced by *S*. *epidermidis* DSM 3095 (Schnell et al. 1992).

It was demonstrated that *bacCH91* occurred in two allelic variants distinguishable by RFLP. The allelic variant I identified in *S*. *aureus* CH-91 was characteristic for the strains of poultry origin and was not present in any of the strains of human origin tested. The isolates of human origin were characterized by allelic variant II, which was also detected in several strains of animal origin. Therefore, the simple RFLP assay may serve as a useful tool for tracing the transmission of staphylococcal strains among hosts.

## Electronic supplementary material

Below is the link to the electronic supplementary material.

ESM 1 (PDF 751 kb)
