# Supplementary material for: Isolation, biochemical characterization, and cloning of a bacteriocin from the poultry-associated Staphylococcus aureus strain CH-91
Source: Appl Microbiol Biotechnol. 2012 Nov 30;97(16):7229–39. doi: 10.1007/s00253-012-4578-y (PMC3724985; doi:10.1007/s00253-012-4578-y)
Supplement: Supplementary file 1 — (PDF 751 kb) [file 253_2012_4578_MOESM1_ESM.pdf]

Supplementary materials

Isolation, biochemical characterization and cloning of a bacteriocin from the poultry-associated *Staphylococcus aureus* strain CH-91

Applied Microbiology and Biotechnology

Benedykt Wladyka, Katarzyna Wielebska, Marcin Wloka, Oliwia Bochenska, Adam Dubin, Pawel Mak

Corresponding author:

Pawel Mak

Department of Analytical Biochemistry, Faculty of Biochemistry, Biophysics and Biotechnology, Jagiellonian University, Gronostajowa 7, 30-387 Krakow, Poland. Phone: +48 12 664 6506. Fax: +48 12 664 6915. E-mail: pawel.mak@uj.edu.pl

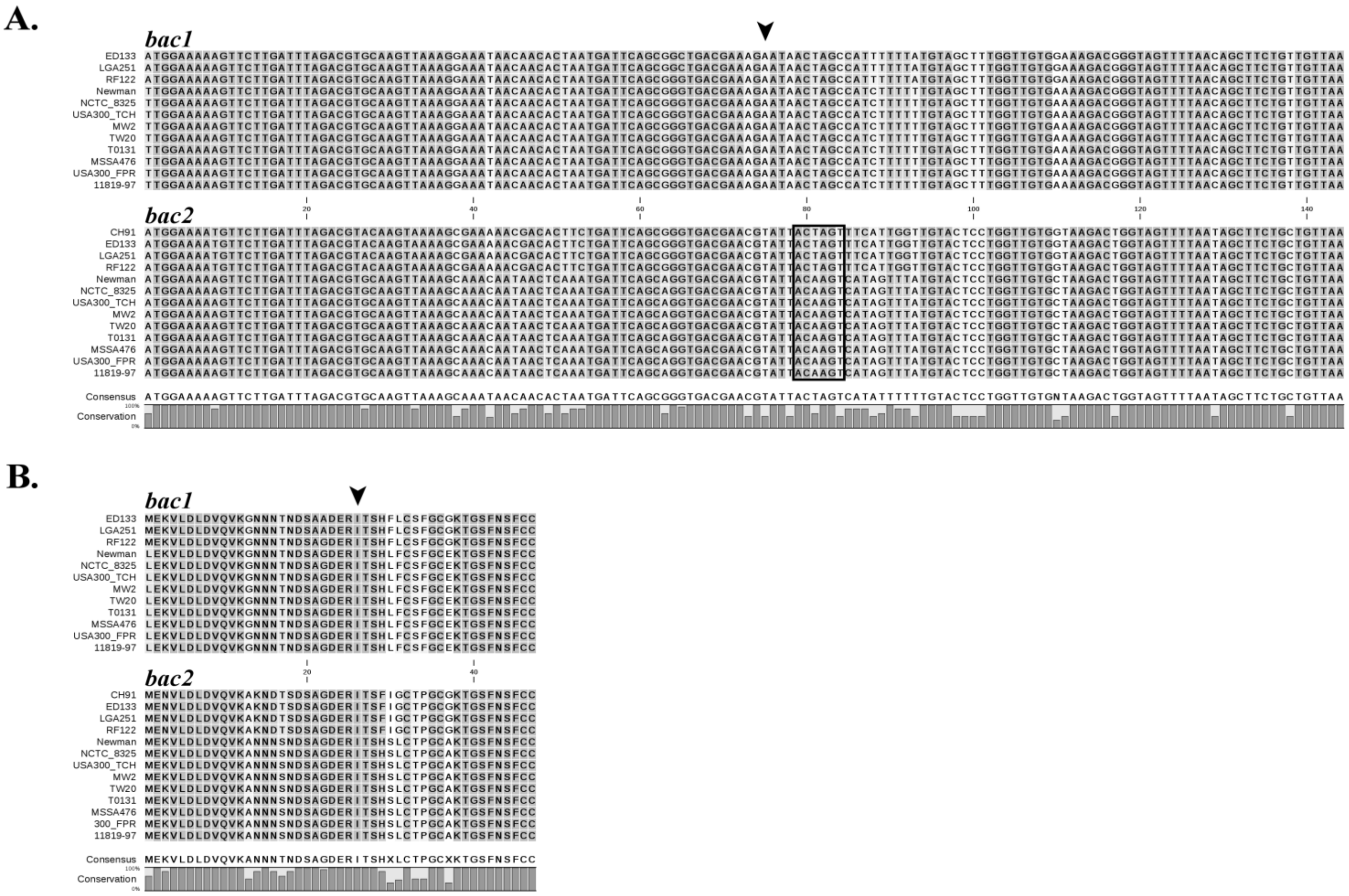

Supplementary Fig. S1 Alignment of the nucleotide (A) and amino acid (B) sequences of BacCH91 variants, as extracted from publically available *S. aureus* genomes. The arrowhead indicates the beginning of the mature form of the peptide. The *bac1* and *bac2* headings refer to the respective ORFs in Fig. 3A. The box indicates the variable nucleotide sequence used in RFLP.

Supplementary Table S1 *S. aureus* strains used for determination of *bac2* gene prevalence

Strain	Host	Geographic origin	Bacteriocin gene	Restriction pattern	Source/reference
MR5	Human	Poland / 1992	+	II	(S6)
N98	Human	Poland / 1995	+	II	(S6)
MR76	Human	Poland / 1992	+	II	(S6)
EMRSA-16	Human	England / 1992	-		(S6)
ZSK987	Human	Poland / 2002	+	II	(S2)
2700	Human	Poland / 1998	-		(S6)
794	Human	Poland / 1997	-		(S6)
3502	Human	Bulgaria / 1998	+	II	(S6)
N39	Human	Poland / 1995	-		(S6)
A005a	Human	Poland / 1992	-		(S6)
2956	Human	Poland / 2001	-		(S6)
1791/97	Human	Poland / 1997	+	II	(S6)
3498	Human	Russia / 1998	+	II	(S6)
3483	Human	Slovenia / 1998	-		(S6)
2688/98	Human	Poland / 1998	-		(S6)
3254	Human	Turkey / 1996	+	II	(S6)
RN4220	Human		+	II	(S4)
Newman	Human		+	II	(S1)
NCTC8325	Human		+	II	NARSA
ATCC_25923	Human		-		ATCC
3248	Human	Czech Republic / 1996	-		(S6)
Ch-91	Poultry	Japan /1967	+	I	(S7)
Ph1	Poultry	Scotland / Unknown	+	I	(S3)
Pa3	Poultry	Scotland / 2006	+	I	(S3)
Ch15	Poultry	Belgium / 1976	+	I	(S3)
Ch8	Poultry	USA / 1999	-		(S3)
Ph2	Poultry	Scotland / Unknown	+	I	(S3)
Pa1	Poultry	Scotland / 1996	-		(S3)
Bu1	Poultry	Scotland / 2008	+	I	(S3)
Ch11	Poultry	Scotland / 2006	+	I	(S3)
Ch10	Poultry	USA / 1999	-		(S3)
Ch5	Poultry	Belgium / 1976	-		(S3)
Pa2	Poultry	Scotland / 1997	-		(S3)
Ch3	Poultry	N. Ireland / 1996-97	-		(S3)
Tu1	Poultry	Scotland / Unknown	-		(S3)
Turkey	Poultry	Poland / 2008	+	II	(S5)
M-121	Poultry	Poland / 2008	-		(S5)
M-122	Poultry	Poland / 2008	-		(S5)
M2_hen	Poultry	Poland / 2008	-		(S5)
MB80/06	Poultry	Poland / 2008	-		(S5)
M1_hen	Poultry	Poland / 2008	+	II	(S5)
M-kon	Horse	Poland / 2008	+	II	Present study
NOTA	Horse	Poland / 2008	-		(S5)
K-Cat	Cat	Poland / 2008	+	I	(S5)
B363	Cat	Poland / 2008	-		(S5)
D97	Dog	Poland / 2007	-		(S5)
D2902	Dog	Poland / 2008	-		(S5)
D89	Dog	Poland / 2007	-		Present study
D92	Dog	Poland / 2007	-		Present study
Szympans	Chimpanzee	Poland / 2008	-		Present study
Tapir	Tapir	Poland / 2008	-		Present study
M-krolik	Rabbit	Poland / 2008	-		Present study
Osiol_P	Donkey	Poland / 2008	+	II	Present study
M6	Cow	Poland / 2009	-		Present study
M11	Cow	Poland / 2009	-		Present study
M13	Cow	Poland / 2009	-		Present study
M25	Cow	Poland / 2009	-		Present study
2527	Cow	Poland / 2008	-		Present study

Supplementary materials references

S1. Duthie ES, Lorenz LL (1952) Staphylococcal coagulase; mode of action and antigenicity. J Gen Microbiol 6:95-107

S2. Kedzierska A, Kapinska-Mrowiecka M, Czubak-Macugowska M, Wojcik K, Kedzierska J (2008) Susceptibility testing and resistance phenotype detection in *Staphylococcus aureus* strains isolated from patients with atopic dermatitis, with apparent and recurrent skin colonization. Br J Dermatol 159:1290-1299

S3. Lowder BV, Guinane CM, Ben Zakour NL, Weinert LA, Conway-Morris A, Cartwright RA, Simpson AJ, Rambaut A, Nubel U, Fitzgerald JR (2009) Recent Human-to-Poultry host jump, adaptation, and pandemic spread of *Staphylococcus aureus*. Proc Natl Acad Sci USA 106:19545-19550

S4. Nair D, Memmi G, Hernandez D, Bard J, Beaume M, Gill S, Francois P, Cheung AL (2011) Whole-genome sequencing of *Staphylococcus aureus* strain RN4220, a key laboratory strain used in virulence research, identifies mutations that affect not only virulence factors but also the fitness of the strain. J Bacteriol 193:2332-2335

S5. Polakowska K, Lis M, Helbin WM, Dubin G, Dubin A, Niedziolka J, Miedzobrodzki J, Wladyka B (2012) Virulence in chicken embryos but not nematode model correlates with a genetic background of *Staphylococcus aureus* strains. Microbes Infect, in press, http://dx.doi.org/10.1016/j.micinf.2012.09.006

S6. Sabat A, Malachowa N, Miedzobrodzki J, Hryniewicz W (2006) Comparison of PCR-based methods for typing *Staphylococcus aureus* isolates. J Clin Microbiol 44:3804-3807

S7. Takeuchi S, Kinoshita T, Kaidoh T, Hashizume N (1999) Purification and characterization of protease produced by *Staphylococcus aureus* isolated from a diseased chicken. Vet Microbiol 67:195-202
